# Complete Genome Sequence of Stenotrophomonas maltophilia Strain CPBW01, Isolated from the Wings of the Colorado Potato Beetle in Xinjiang, China

**DOI:** 10.1128/MRA.00460-20

**Published:** 2020-06-18

**Authors:** Wei-Nan Kang, Kai-Yun Fu, Wen-Chao Guo, Guo-Qing Li

**Affiliations:** a Education Ministry Key Laboratory of Integrated Management of Crop Diseases and Pests, College of Plant Protection, Nanjing Agricultural University, Nanjing, China; b Institute of Plant Protection, Xinjiang Academy of Agricultural Sciences, Urumqi, China; c Key Laboratory of Integrated Management of Harmful Crop Vermin of China North-Western Oasis, Ministry of Agriculture, Urumqi, China; d Institute of Microbiological Application, Xinjiang Academy of Agricultural Science, Urumqi, China; Indiana University, Bloomington

## Abstract

Bacteria of the genus *Stenotrophomonas* are opportunistic and have been documented in the guts of several insect species. Here, we present the complete genome sequence of S. maltophilia strain CPBW01, isolated from the wings of the Colorado potato beetle, Leptinotarsa decemlineata, collected from potato fields in Urumqi (43.71N, 87.39E), Xinjiang, China.

## ANNOUNCEMENT

Bacteria of the genus *Stenotrophomonas* are found throughout the environment (1). These bacteria have been documented in the guts of several insect species, including the Colorado potato beetle (CPB), Leptinotarsa decemlineata (2–5). However, the *Stenotrophomonas* species collected from insects have not been identified. Given that *Stenotrophomonas* spp. can metabolize a large range of organic compounds (1), we hypothesized that the *Stenotrophomonas* species in CPBs can aid their host to metabolize solanine, the major secondary compound in potatoes. Since CPB wings are easily dissected, we cut the wings using surgical scissors at the point above the wing base to avoid collecting any muscle tissue. We washed the collected wings with sterile water 5 times and cultured the collection with solanine as the unique source of carbon in minimal medium (Bushnell Haas medium). As a result, we isolated *Stenotrophomonas* strain CPBW01 from the wings of the beetles collected from the potato fields. The bacteria cultured on solid minimal medium with solanine gave the appearance of single colonies.

We sequenced the genome of CPBW01 using third-generation sequencing technology with the following steps. A total of 15 μl of the bacterial minimal medium culture with solanine as the unique carbon source was added into 1,200 ml Luria-Bertani medium with ampicillin (0.05 mg/ml) and kanamycin (0.05 mg/ml). Genomic DNA was extracted from the culture, which was grown for 23 h at 30°C under agitation (200 rpm), using the Qiagen genomic DNA extraction kit (catalog number 13323) according to the standard operating procedure provided by the manufacturer. The extracted DNA was detected with the NanoDrop One UV-visible (UV-Vis) spectrophotometer (Thermo Fisher Scientific, USA) for DNA purity (optical density ratio at 260/280 nm [OD_260_/OD_280_] range, 1.8 to 2.0; OD_260_/OD_230_ range, 2.0 to 2.2). The Qubit 3.0 fluorometer (Invitrogen, USA) was then used to accurately quantify the DNA. Long DNA fragments were extracted from the qualified sample using the BluePippin system (Sage Science, USA). The long DNA fragments were repaired, and the DNA ends were prepared for adapter attachment. The sequencing adapters supplied in the SQK-LSK109 kit were attached to the DNA ends. Finally, the Qubit 3.0 fluorometer was used to quantify the size of the library fragments. The DNA library was loaded onto a flow cell, transferred to the Nanopore GridION X5/PromethION sequencer (Oxford Nanopore Technologies, UK), and sequenced.

The BGISEQ-500 platform was used for sequencing, and the raw sequence data are available in the SRA under accession number SRX7441137. In total, 3,040,424,894 bp of raw data were obtained, which included 159,420 reads, with a mean length of 19,071 bp and an *N*_50_ value of 26,557 bp. The longest read was 171,958 bp. After elimination of adaptor sequences and low-quality reads, the high-quality reads were assembled into contigs using Canu 1.7.11 (https://github.com/marbl/canu) and corrected using Pilon 1.22 (https://github.com/broadinstitute/pilon) and second-generation sequencing data. Then, the assembled fragments were further aligned, scaffolded, and checked to produce a circular chromosome, using Canu, followed by Circulator 1.2.0 (parameter: fixstart). The chromosome was confirmed to be circular by the Circlator software 1.2.0. For CPBW01, 4,444,327 bp of clean data filtered from raw reads were generated to reach a sequencing depth of about 634-fold, with a GC content of 66.55%.

The Prodigal software 2.6.3 (https://github.com/hyattpd/prodigal) was used to predict the gene models. All gene models were then subjected to a BLAST search against the KEGG (http://www.genome.jp/kegg/) (6), COG (http://www.ncbi.nlm.nih.gov/COG) (7), and GO (20180828 http://geneontology.org/) databases on 9 August 2019 to perform functional annotation. The assembled sequence contains 3,962 protein-coding genes, 13 rRNAs, 75 tRNAs, and 1 genomic island.

The average nucleotide identity (ANI) was calculated using an algorithm described by Yoon et al. (8) with the Web service EzBioCloud. The genome of strain CPBW01 is 98.08% identical to that of S. maltophilia strain ISMMS2, 91.10% identical to that of S. pavanii strain LMG 25348, and 79.02% identical to that of S. nitritireducens strain 2001. Given that the ANI cutoff is 94% for delineating *Stenotrophomonas* species (9), our data demonstrate that CPBW01 is a strain of S. maltophilia. Comparative genomic sequence analysis between strains CPBW01 and ISMMS2 from different environments will allow a more comprehensive characterization of this bacterium.

### Data availability.

The nucleotide sequence is available at GenBank under the accession number NZ_CP047310.1 and BioSample number SAMN13642261. The raw sequence reads have been deposited in the NCBI Sequence Read Archive under accession number SRP238799.
